# Draft Genome Sequence and Biosynthetic Potential of the Newly Described Strain Longimicrobium terrae CB-286315^T^

**DOI:** 10.1128/MRA.00512-20

**Published:** 2020-06-11

**Authors:** Marina Sánchez-Hidalgo, Javier Pascual, Ignacio González, Olga Genilloud

**Affiliations:** a Department of Microbiology, Fundación MEDINA, Granada, Spain; Georgia Institute of Technology

## Abstract

Longimicrobium terrae CB-286315^T^, the first member of the family *Longimicrobiaceae*, was isolated at the Sierra de Tejeda, Almijara and Alhama Natural Park, Spain, using a diffusion sandwich system (DSS). We present the draft genome sequence of this strain, which comprised 6,886,230 bp. A total of 12 putative biosynthetic gene clusters were predicted, including ribosomally synthesized and posttranslationally modified peptides (RiPPs), polyketides, and nonribosomal peptides.

## ANNOUNCEMENT

Longimicrobium terrae CB-286315^T^ was isolated from a Spanish Mediterranean forest soil using the diffusion sandwich system (DSS) (1). This bacterium was identified as the type strain of a novel genus and species, Longimicrobium terrae gen. nov., sp. nov., within the phylum *Gemmatimonadetes* (1).

The genome of strain CB-286315^T^ was *de novo* sequenced using PacBio and Illumina technologies. Unless otherwise noted, default parameters were used for all software.

Bacterial cells recovered from solid plates of R2A (DSMZ medium 830) were used to extract the DNA following the protocol recommended by Pacific Biosciences. Quality control of the DNA was determined photometrically (NanoDrop One; Thermo Scientific) and fluorometrically (PicoGreen; Promega).

PacBio sequencing was performed by Lifesequencing (Valencia, Spain). The DNA was sheared to produce ∼20-kb insert size libraries using the BluePippin size selection system (protocol no. 100-286-000-07). The mean size distribution was 27,889 bp.

The sample was sequenced using P6-C4 chemistry (Pacific Biosciences), MagBead loading, stage start, and a data acquisition time of ≥4 h. A total of 1,572 Gb of raw data were obtained. The optimal sequences (minimal polymerase read length, 500 bp; minimal polymerase read quality, 0.8; minimal subread length, 500 bp) were *de novo* assembled using HGAP3.0 (SMRT analysis version 2.3.0, patch 4) with a 6,000-bp minimum seed read length, resulting in 8 contigs and 6,836,795 bp. Additionally, the Circlator tool (2) yielded 4 noncircularized contigs and 6,877,379 bp.

Illumina short read libraries (200 to 300 bp) were prepared with the Nextera XT kit (Illumina). Sequencing was performed using 150-bp paired-end reads on the MiSeq platform at the Center for Scientific Instrumentation of the University of Granada in Spain. A total of 15,188,663 paired sequences were generated with a theorical coverage of >300×. Quality control, trimming, and assembly were performed with Orione tools (3). We used SPAdes (4) and Velvet (5) assemblers with parameters determined by VelvetOptimiser version 2.2.5 (http://bioinformatics.net.au/software.velvetoptimiser.shtml). The resulting sequences were merged using CISA (6), yielding 795 contigs (*N*_50_, 12,021 bp).

The Illumina contigs were mapped to the PacBio sequences using Geneious 9.1.2, generating 6,886,230 bp and four contigs (contig 1, 3,950,089 bp; contig 2, 2,920,167 bp; contig 3, 8,132 bp; contig 4, 7,842 bp). The genomic GC content was 69.4%.

The genome was analyzed with antiSMASH version 5.1.2 (7) to identify potential secondary metabolite biosynthetic gene clusters (BGCs). Twelve putative BGCs were predicted (Table 1). Two of the nonribosomal peptide synthetase (NRPS) BGCs showed 60% and 100% similarity with the ralsolamycin and bicornutins BGCs (8, 9), respectively (Table 1). Interestingly, the predicted ribosomally synthesized and posttranslationally modified peptides (RiPPs) did not show homologies with currently known compounds. Further characterization of such pathways and metabolites could be of great interest for identifying novel compounds with biomedical activities from a novel and unexplored taxon.

**TABLE 1:** AntiSMASH prediction of secondary metabolite biosynthetic gene clusters in the genome sequence of Longimicrobium terrae CB-286315^T^

Cluster no.	Contig no.	Type^*a*^	Nucleotide position (start–stop)	Most similar known cluster	MIBiG^*b*^ accession no.	Similarity (%)
1	1	Phosphonate, NRPS, T1PKS	478782–568034	Lysobactin	BGC0000385	2
2	1	NRPS, T1PKS	955517–1107825	Crochelin A	BGC0002001	15
3	1	Lassopeptide	1879461–1901676	ND^*c*^	ND	ND
4	1	NRPS	2337548–2386467	ND	ND	ND
5	1	Lanthipeptide, NRPS	2776448–2920167	Ralsolamycin	BGC0001363	60
6	2	NRPS, T1PKS	620932–693866	Ralsolamycin	BGC0001363	60
7	2	NRPS, T1PKS	807797–914522	Microsclerodermin M	BGC0001019	18
8	2	Bacteriocin	1004003–1014929	ND	ND	ND
9	2	Lanthipeptide	1694604–1719592	ND	ND	ND
10	2	Lanthipeptide	2770211–2794619	ND	ND	ND
11	2	Terpene	2946314–2971366	ND	ND	ND
12	2	NRPS	3926585–3950089	Bicornutins A1/A2	BGC0001135	100

a NRPS, nonribosomal peptide synthetase; T1PKS, type 1 polyketide synthase.

b MIBiG, Minimum Information about a Biosynthetic Gene cluster (10).

c ND, not determined.

### Data availability.

This whole-genome shotgun project has been deposited in GenBank under the accession no. JABDTL000000000. The version described in this paper is the first version, JABDTL010000000. The raw sequencing reads have been deposited in the SRA under the accession no. SRR11780528 (PacBio) and SRR11794304 (Illumina) and are associated with BioProject no. PRJNA629516 and BioSample no. SAMN14777635.
